# Spatial and Temporal Dynamics in the Ionic Driving Force for GABA_A_ Receptors

**DOI:** 10.1155/2011/728395

**Published:** 2011-06-27

**Authors:** R. Wright, J. V. Raimondo, C. J. Akerman

**Affiliations:** Department of Pharmacology, University of Oxford, Mansfield Road, Oxford OX1 3QT, UK

## Abstract

It is becoming increasingly apparent that the strength of GABAergic synaptic transmission is dynamic. One parameter that can establish differences in the actions of GABAergic synapses is the ionic driving force for the chloride-permeable GABA_A_ receptor (GABA_A_R). Here we review some of the sophisticated ways in which this ionic driving force can vary within neuronal circuits. This driving force for GABA_A_Rs is subject to tight spatial control, with the distribution of Cl^−^ transporter proteins and channels generating regional variation in the strength of GABA_A_R signalling across a single neuron. GABA_A_R dynamics can result from short-term changes in their driving force, which involve the temporary accumulation or depletion of intracellular Cl^−^. In addition, activity-dependent changes in the expression and function of Cl^−^ regulating proteins can result in long-term shifts in the driving force for GABA_A_Rs. The multifaceted regulation of the ionic driving force for GABA_A_Rs has wide ranging implications for mature brain function, neural circuit development, and disease.

## 1. Introduction

GABA_A_ receptors (GABA_A_Rs) are the principal mediators of fast synaptic inhibition in the brain. These receptors differ from most ligand-gated ion channels in that their reversal potential (*E*_GABA_) is close to the resting membrane potential of neurons. Consequently, GABA_A_Rs have the capacity to exhibit a different form of dynamics whereby small changes to the driving force of the underlying anionic currents can lead to significant changes in the nature and strength of GABA_A_R-mediated transmission. For instance, if *E*_GABA_ is more negative than the resting membrane potential GABA_A_R activation will result in membrane hyperpolarisation and inhibition. If *E*_GABA_ is more positive than the resting membrane potential however, stimulating GABA_A_Rs will result in a combination of membrane depolarization and shunting inhibition. GABA_A_R activation is only excitatory if *E*_GABA_ is positive enough to increase the probability of action potential generation. 

**Figure 1:**
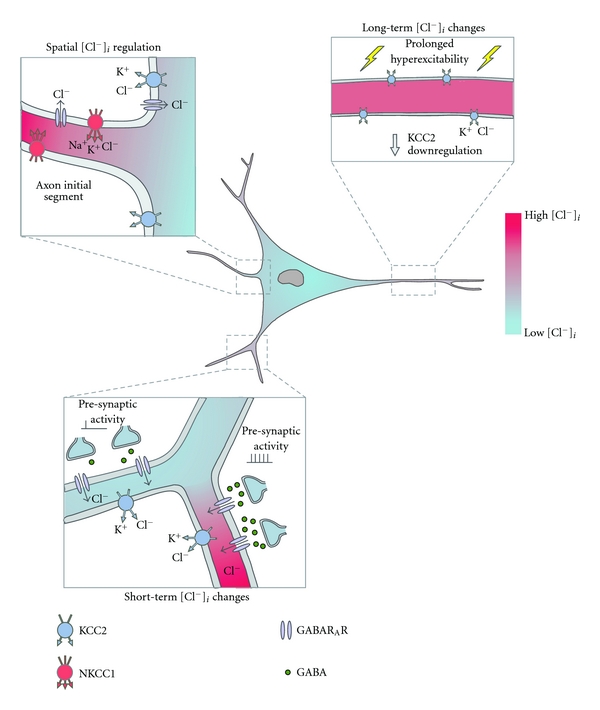
[Cl^−^]*_i_* and the associated driving force for GABA_A_Rs can be subject to spatial and activity-dependent temporal variations. The upper left panel shows an example of spatially regulated [Cl^−^]*_i_*. It has been reported that low levels of KCC2 expression within the axon initial segment enable NKCC1 to maintain relatively high levels of [Cl^−^]*_i_* compared to the soma (indicated by the red colour inside the cell) [22–24]. This can generate a depolarising Cl^−^ driving force for GABA_A_Rs within the axon [21–23]. The lower left panel shows an example of short-term [Cl^−^]*_i_* loading within dendritic branches. Cl^−^ influx associated with low-level GABA_A_R activity is dealt with by Cl^−^ regulation mechanisms (left-hand dendritic branch). However, during periods of intense GABA_A_R activation, if *E*_Cl^−^_ is hyperpolarised with respect to the membrane potential, high levels of Cl^−^ influx via GABA_A_Rs can lead to localised increases in [Cl^−^]*_i_* and consequently depolarising shifts in *E*_GABA_ (right-hand dendritic branch) [25, 26]. The upper right panel illustrates an example of long-term [Cl^−^]*_i_* changes. Certain patterns of neural activity within mature neurons (e.g., repetitive coincidental pre- and postsynaptic spiking or prolonged postsynaptic spiking, interictal-like activity) can lead to a downregulation in KCC2 activity, resulting in long-term increases in [Cl^−^]*_i_* [10, 27, 28].

The best described example of *E*_GABA_ modulation occurs during early development when neurons in the hippocampus and other brain structures have been shown to undergo a shift in the ionic driving force for GABA_A_Rs from depolarising to hyperpolarising [1–4]. This change is the result of a developmental decrease in the levels of intracellular chloride ([Cl^−^]*_i_*), brought about by the increased contribution of the K^+^-Cl^−^ cotransporter, KCC2, which extrudes Cl^−^, compared to the Na^+^-K^+^-2Cl^−^ cotransporter, NKCC1, which normally functions to raise [Cl^−^]*_i_* [5]. Changes to [Cl^−^]*_i_* and GABA_A_R-mediated currents have also been described as a result of neural trauma [6–17]. Since the reports that the Cl^−^ driving force for GABA_A_Rs is altered during development and in particular CNS disorders, there has been further careful examination of how neurons regulate [Cl^−^]*_i_*. This work confirms that [Cl^−^]*_i_* and the associated ionic driving force for GABA_A_Rs cannot be thought of as a fixed parameter. Rather, sophisticated mechanisms impact how Cl^−^ is regulated in space and time, such that [Cl^−^]*_i_* can vary between cells, within different parts of the same cell, and as a function of the history of the cell and the network in which it resides. Appreciating these mechanisms is important for understanding GABAergic signalling, not only in the mature nervous system, but also during neural circuit formation and in the context of CNS disorders. The diagram in Figure 1 provides an outline for this review by illustrating three ways in which the ionic driving force for GABA_A_Rs may exhibit differences. We will focus on recent work that has examined how spatial properties of neurons have been linked to differences in [Cl^−^]*_i_* and how activity-dependent mechanisms can generate both short- and long-term changes in [Cl^−^]*_i_*. In doing so, we will also discuss the potential functional consequences of spatial and temporal differences in driving force for GABA_A_Rs.

## 2. Spatial Variations in *E*_GABA_

Over recent years it has become increasingly apparent that the notion of universally hyperpolarising *E*_GABA_ in mature neurons of the CNS is a misleading one. *E*_GABA_ can vary across different types of neurons and this leads to different actions of GABA_A_R postsynaptic potentials (GPSPs) depending on the cell type in question [18–20]. For example, fast spiking inhibitory interneurons in the cortex and amygdala exhibit a considerably more depolarised *E*_GABA_ than neighbouring pyramidal cells, which may contribute to differences in the excitability of these two cell types [18]. What has also come to be appreciated is the fact that as well as intercellular variability, *E*_GABA_ can show *intracellular* differences. One of the most prominent examples involves the axon initial segment (AIS). Here, the *E*_GABA_ of inputs from axoaxonic (or Chandelier) cells tend to be significantly more positive than the *E*_GABA_ of separate GABAergic inputs targeting the soma [21–23] (see Figure 1). Axonal *E*_GABA_, as determined in three studies, was found to be between 6 and 22 mV more positive than somatic *E*_GABA_ [21–23]. Such within-cell variations in *E*_GABA_ have been linked to the differential distribution of Cl^−^ cotransporter proteins. Immunogold labelling of KCC2 in hippocampal pyramidal and dentate gyrus cells has shown that the levels of this transporter are severalfold higher in the soma compared to the AIS, with local KCC2 densities at the plasma membrane of the AIS at around 6% the level of somatic KCC2 [23, 24]. NKCC1-null cells, or cells treated with bumetanide, do not exhibit axosomatic [Cl^−^]*_i_* gradients, which indicates that NKCC1 is key to maintaining the higher *E*_GABA_ values recorded at the AIS [22]. 

The degree of differences in *E*_GABA_ between axon and soma may vary across different cell types and whether the resultant effect of an axoaxonic GABAergic input to a neuron is depolarising, hyperpolarising, inhibitory, or even excitatory is still not clear [29, 30]. The location of the AIS is close to the proposed site of action potential initiation and thus one might predict that if axoaxonic inputs are indeed depolarising these could help promote action potential initiation [21]. However, despite numerous studies [21–23, 30, 31] there is limited evidence that GABA_A_R synapses formed by axoaxonic cells at the AIS are able to trigger action potentials in the postsynaptic neuron. It is important to note that, even with depolarising driving forces, GABA_A_R synapses may still exert strong inhibitory effects via their shunting action upon excitatory currents [32]. Consequently, whether AIS GABA_A_R synapses are capable of evoking excitatory responses in pyramidal cells is still an open question and one that will be dependent on factors such as the number and relative timing of GABAergic and glutamatergic inputs, the magnitude of the GABA_A_R conductance and whether or not the depolarising actions persist beyond the shunting effect [33].

Local [Cl^−^]*_i_* differences can also be found between the soma and dendrites of several types of neurons [22, 34, 35]. For example, [Cl^−^]*_i_* has been shown to be higher and more depolarising in the dendrites than in the soma of certain ON-type retinal bipolar cells, a difference that underlies the receptive field properties of these neurons [34]. Numerous other studies, utilising a wide array of different techniques and preparations, have reported considerable variation in the strength and direction of somatodendritic Cl^−^ gradients [22, 35–39]. These differences can typically be explained by compartment specific expression of Cl^−^ transporter proteins regulated as a function of development, cell type, and brain region [34, 40]. However, it is worth remembering that because the degree of phasic and tonic GABA_A_R activity can itself influence [Cl^−^]*_i_*, and can also vary significantly between different experimental preparations, this may affect estimates of [Cl^−^]*_i_* [41].

In a recent study, Földy et al. [42] discovered intracellular Cl^−^ regulation on an even more spatially refined scale and via a mechanism involving Cl^−^ regulators other than transport proteins. The authors examined the conductance and current-rectification properties of two types of GABAergic input onto the same perisomatic region of CA1 pyramidal neurons. Their recordings revealed that GABA_A_R currents at synapses receiving presynaptic input from parvalbumin-expressing fast-spiking basket cells (PVBCs) are selectively modulated by the voltage-gated Cl^−^ channel ClC-2. ClC-2 is found in the soma of pyramidal neurons and is an inwardly rectifying channel, which is activated by neuronal hyperpolarisation and allows Cl^−^ to flow out of the cell more easily than into it [43, 44]. ClC-2 activity was found to be strongly associated with PVBC synapses, in contrast to neighbouring synapses formed by cholecystokinin-expressing basket cells (CCKBCs). As a consequence, rates of Cl^−^ extrusion following intense GABA_A_R activity were found to be significantly faster at PVBC synapses. This is supported by Rinke et al. [45], who reported that neurons from mice lacking the ClC-2 channel show reduced rates of Cl^−^ removal and by the fact that the resting *E*_GABA_ at PVBC synapses is significantly lower than at CCKBC synapses [42]. The authors suggest that the presence of somatic CLC-2 and its contribution to Cl^−^ regulation could play an important role in preventing potentially detrimental increases in [Cl^−^]*_i_* during periods of intense firing by soma targeting PVBCs [42]. As Földy et al. point out, their findings could be partly explained at a compartmental level, as the somatodendritic distribution of PVBC and CCKBC synapses does show some differences. Nevertheless, these recent studies have advanced our appreciation of Cl^−^ regulation by showing that, as well as being nonuniform across different neuronal compartments, *E*_GABA_ may vary between individual synapses within the same compartment. Thus, even assigning *E*_GABA_ to certain spatial regions of a cell may be an oversimplification and instead it could be more appropriate to consider *E*_GABA_ in terms of a particular input to a postsynaptic cell [46]. 

## 3. Short-Term Temporal Changes in *E*_GABA_

In addition to spatial variation, *E*_GABA_ can also show rapid temporal changes within individual cells (see Figure 1). It is well known that responses to intense GABA_A_R activation can change from being hyperpolarising to depolarising in less than a second [36, 47, 48]. Such biphasic responses are now generally thought to represent a depolarising shift in *E*_GABA_, caused by the differential collapse of the opposing concentration gradients of Cl^−^ and HCO_3_^−^ [25, 26, 49]. GABA_A_Rs are approximately five times more permeable to Cl^−^ than HCO_3_^−^ [50]. Therefore at rest, *E*_GABA_ (typically −75 mV) is much closer to the very negative Cl^−^ reversal (*E*_Cl^−^_; typically −85 mV) than the considerably more positive HCO_3_^−^ reversal (*E*_HCO_3_^−^_; typically −20 mV) [51]. During intense activation of GABA_A_Rs however, rapid Cl^−^ influx exceeds Cl^−^ extrusion mechanisms and a breakdown in the Cl^−^ gradient occurs. An equivalent collapse of the HCO_3_^−^ gradient is prevented by the activity of intra- and extracellular carbonic anhydrases, which use CO_2_ as a substrate to rapidly regenerate intracellular HCO_3_^−^. As a result, with continued GABA_A_R activation *E*_GABA_ shifts toward the more positive *E*_HCO_3_^−^_, and this accounts for the depolarising phase of the biphasic response [25, 52]. Indeed, by blocking carbonic anhydrase with the drug acetazolamide, the depolarising response to strong GABA_A_R activation is prevented [26]. Interestingly, a recent paper argues that this GABA elicited depolarisation is paradoxically accentuated by the activity of the electroneutral cotransporter KCC2 [53]. Following the GABA_A_R—mediated accumulation of intracellular Cl^−^, this leads to an accelerated extrusion of both Cl^−^ and K^+^ by KCC2. Provided this extrusion of K^+^ occurs within a large enough neuronal population, the increase in extracellular K^+^ can result in inward K^+^ currents that further depolarise the cell membrane [49, 53]. 

The shifts in *E*_GABA_ that are associated with intense GABA_A_R activation are transient, such that once GABA_A_R activity subsides [Cl^−^]*_i_* returns to baseline levels within seconds or minutes [25, 54]. Any factor that affects the rate of Cl^−^ accumulation during GABA_A_R activation will affect how rapidly and by how much *E*_GABA_ shifts. For instance, the volume of the neuronal compartment that receives the GABAergic input is one important parameter. For a given amount of synaptic GABA_A_R stimulation and its accompanying Cl^−^ influx, smaller postsynaptic volumes will result in relatively larger increases in [Cl^−^]*_i_*. As a result, dendritic compartments are more susceptible to Cl^−^ accumulation (and hence depolarising shifts in *E*_GABA_) than the soma [25, 41]. In a theoretical paper, Qian and Sejnowski [55] utilised this reasoning to suggest that GABA_A_R-mediated inhibition is likely to be ineffective on dendritic spines. Due to their minute volume, even small amounts of Cl^−^ influx would result in a local increase in [Cl^−^]*_i_* that would rapidly depolarise *E*_GABA_. Consistent with this idea, it has since been confirmed that most GABAergic synapses are localised to dendritic shafts as opposed to spines [56, 57]. As described above, another important factor that affects Cl^−^ accumulation during GABA_A_R activity is the presence, affinity and capacity of carbonic anhydrase. Given the significance of cell volume and carbonic anhydrase activity, it is perhaps not surprising therefore that different cell types might differ in their susceptibility to Cl^−^ accumulation. For example, Lamsa and Taira [54] found that 10–100 Hz stimulation trains produce depolarising switches in the *E*_GABA_ of interneurons of the CA3 stratum pyramidale and stratum oriens regions, but were unable to evoke similar shifts in CA3 pyramidal neurons. 

In order to evoke the depolarising shifts in *E*_GABA_ described above, intense GABA_A_R activation has been elicited either by exogenous application of GABA_A_R agonists or high-frequency stimulation of GABAergic afferents. Evidence that such short-term changes in *E*_GABA_ could occur in vivo have come from studies of hyperactive network activity patterns, such as those generated in experimental models of epilepsy. It is believed that the intense activation of GABA_A_Rs that occurs during seizures can cause rapid Cl^−^ accumulation [58–62]. Indeed, the resultant erosion of GABA_A_R-mediated inhibition serves to initiate or exacerbate the hyperexcitability that is characteristic of epileptiform events [63]. Beyond seizure activity, it is currently an open question as to what range of physiologically relevant activity patterns could lead to short-term changes to *E*_GABA_, and what the functional impact upon circuit function might be. Nevertheless it is interesting that levels of [Cl^−^]*_i_* accumulation would appear to increase linearly with the intensity and number of stimulations, and even relatively weak stimulation can produce small changes in [Cl^−^]*_i_* [62, 64].

Another area that has yet to be fully investigated concerns how short-term activity-dependent shifts in *E*_GABA_ might affect developing neurons. It has already been established that during the first two weeks of postnatal life, rat hippocampal neurons express low levels of intracellular carbonic anhydrase and therefore do not exhibit the HCO_3_^−^ dependent GABA_A_R depolarisation that mature neurons display following high-frequency synaptic activity [52, 65]. And it seems likely that other properties of immature neurons would contribute to a different susceptibility to activity-driven Cl^−^ accumulation or depletion. These include the higher resting [Cl^−^]*_i_* observed in young neurons, plus different expression patterns of Cl^−^ transporter proteins [5, 66, 67] and Cl^−^ permeable channels [45, 68]. One area for future work will be to dissect the role that short-term activity-driven shifts in *E*_GABA_ play in both the normal and abnormal development of neural circuits.

## 4. Long-Term Temporal Changes in *E*_GABA_

As we saw in the previous section, brief periods of high-intensity synaptic activity can give rise to short-term changes in the ionic driving force for GABA_A_Rs. There are however, a growing number of examples whereby different forms of neural activity can give rise to more enduring changes in *E*_GABA_ and the underlying [Cl^−^]*_i_* (see Figure 1). Many of these long-term changes in *E*_GABA_ are linked to hyperexcitability disorders, such as epilepsy [8–10, 15, 16, 69] and neuropathic pain [7, 17, 70, 71] and have also been observed in other cases of neuronal trauma such as neural axotomy [11], ischemia [12, 13], and in spasticity models following spinal cord injury [14]. Yet similar long-lasting changes to *E*_GABA_ have also been reported in healthy tissue following certain neural activity patterns [27, 28, 72–78]. In order to better understand these shifts in inhibitory plasticity and their roles in both healthy and pathological neural signalling, a number of studies have begun to investigate the underlying mechanisms behind long-term activity-dependent changes to *E*_GABA_. 

One of the first such investigations focused on the effects of epileptiform activity in hippocampal slices. Here, interictal activity, brought on with low Mg^2+^ conditions, switched the driving force of GPSPs from hyperpolarising to depolarising in CA1 pyramidal cells [10]. This depolarising shift in *E*_GABA_ corresponded to a significant reduction in KCC2 mRNA and protein levels, as well as an increased rate of removal of the Cl^−^ cotransporter from the cell membrane [10]. Similar reductions in KCC2 mRNA and protein could also be observed following *in vivo *kindling [9], and in both cases the activity-led downregulation in KCC2 expression was found to be dependent on BDNF signaling. Scavenging endogenous BDNF with TrkB receptor bodies, or pharmacologically inhibiting TrkB, blocked the activity-induced downregulation of KCC2 and thus suggests that the mechanism involves a BDNF-TrkB signalling interaction [9, 10]. A similar role for BDNF-TrkB signalling has since been reported in the context of positive shifts in *E*_GABA_ and reductions in KCC2 levels within neuropathic and inflammatory pain models [7, 79, 80], suggesting that endogenous BDNF signalling may be a common mechanism by which KCC2 is downregulated during aberrant neural activity. 

Aside from pathological models, changes to *E*_GABA_ and the resultant inhibitory plasticity have also been investigated in the context of more normal physiological signalling. For example, periods of paired pre- and postsynaptic spiking activity have been found to lead to a small but persistent depolarising shift in the postsynaptic *E*_GABA_, of around 3-4 mV in mature rat hippocampal pyramidal neurons [27]. Such long-term depolarising shifts in *E*_GABA_ have also been observed following sustained periods of postsynaptic spiking at frequencies of 10–20 Hz, without presynaptic activity [28]. In both cases the reduction in GABAergic synaptic inhibition was linked to a sustained decrease in KCC2 transporter activity, which in turn was dependent upon Ca^2+^ signalling via L-type Ca^2+^ channels [27, 28]. Further investigation revealed that the activity-dependent downregulation in KCC2 activity requires protein kinase C (PKC) activity, although other studies have since shown that PKC can promote KCC2 activity by stabilising the cotransporter at the membrane surface [81]. Interestingly, Wang et al. [77] recorded from neurons of the subthalamic nucleus and showed for the first time that GABAergic plasticity could be induced in either direction, either generating hyperpolarising or depolarising shifts in GPSPs depending on the degree of rebound spiking activity. Based on further pharmacological experiments the authors proposed that the level of Ca^2+^ increases may be key to determining the nature of GABA_A_R plasticity, with large increases being associated with negative shifts in *E*_GABA_ and small rises in Ca^2+^ leading to positive shifts in *E*_GABA_ [77].

Developmental stage would appear to be critically important for determining the nature and mechanism underlying long-term changes in the ionic driving force for GABA_A_Rs. Within mature cells, such activity-driven changes appear to work by targeting KCC2 and reducing the activity and/or expression of this transporter. This raises the question of what happens within younger neurons when *E*_GABA_ is still depolarising and levels of KCC2 protein are typically low. Are immature neural networks subject to similar activity-dependent long-term [Cl^−^]*_i_* alterations and if so, what are the downstream targets for such mechanisms? To date only a small number of studies have addressed this question directly but already an interesting dichotomy between mature and immature GABA_A_R plasticity regulation is beginning to emerge. For example, as already mentioned, in mature hippocampal slices when *E*_GABA_ is hyperpolarising, application of seizure models has been linked to a depolarising shift in *E*_GABA_ values coupled with a downregulation in KCC2 expression [9, 10]. By contrast, in neonatal hippocampal slices, seizure activity induced by kainic acid have been found to result in either a depolarising [8, 82] or a hyperpolarising shift in *E*_GABA_ [83, 84]. In the latter cases, more negative *E*_GABA_ values have been linked to an increase in KCC2 expression and activity [84, 85]. 

Such variations may be partially due to the type of seizure model used, yet similar age-dependent differences can also be found in other examples of activity-driven *E*_GABA_ changes. For example, a protocol of paired pre- and postsynaptic spiking at 5 Hz, which has been shown previously to elicit depolarising *E*_GABA_ shifts in the mature rat hippocampus [27], actually produces a long-term hyperpolarising shift when applied to the same neurons earlier in development [72, 78]. Rather than targeting KCC2, the hyperpolarising shift in *E*_GABA_ in immature neurons occurs via a downregulation of the NKCC1 transporter, which results in a decrease in [Cl^−^]*_i_* [72]. As in mature systems, the direction of such GABA_A_R shifts in developing neurons can change according to the nature of the stimulus. While paired pre- and postsynaptic spiking at 5 Hz hyperpolarised *E*_GABA_ at developing synapses, stimulation at higher frequencies (20–50 Hz) produces the opposite effect and results in *E*_GABA_ values that are more depolarising [78]. This long-term shift in driving force for GABA_A_Rs induced by high-frequency paired spiking was again mediated through the regulation of NKCC1 activity and required increases in intracellular Ca^2+^, either via L-type Ca^2+^ channels or from internal Ca^2+^ stores [78]. Thus, while spiking-induced activation of L-type Ca^2+^ channels can result in a similar increase in [Cl^−^]*_i_* in both mature and immature hippocampal neurons, the frequency at which it occurs, and the Cl^−^ cotransporter that is targeted, varies according to developmental stage. A similar phenomenon has been observed following periods of experimentally induced postsynaptic spiking. Prolonged spiking at 20 Hz has been shown to lead to depolarising shifts in *E*_GABA_ within both the mature [28] and immature hippocampus [86]. Yet while the underlying mechanism has been linked to Ca^2+^ influx and KCC2 downregulation in mature cells [28], in younger neurons the change in *E*_GABA_ would appear to occur via a different mechanism. Here, postsynaptic spiking is believed to trigger increases in Na^+^-K^+^-ATPase activity, which alters the balance of Na^+^ across the membrane. This shifts the thermodynamic equilibrium of NKCC1 and results in an increase in the rate at which Cl^−^ is transported into the cell [86]. Thus, just as spatial regulation of *E*_GABA_ can show age-specific variation, the mechanisms underlying long-term activity-dependent changes in *E*_GABA_ can also vary according to the developmental stage of the neuron.

What are the functional consequences of such long-term alterations to *E*_GABA_? Changes to [Cl^−^]*_i_* and the resultant Cl^−^ driving force for GABA_A_Rs have been speculated to be involved in long-term potentiation (LTP)—the best studied form of persistent change in synaptic efficacy. In a recent study, Ormond and Woodin [73] found that paired stimulation protocols designed to induce glutamatergic LTP in mature rat hippocampal slices also produced depolarising shifts in *E*_GABA_. The resultant reduction in the strength of inhibitory synaptic input occurred in parallel to “classical” LTP at glutamatergic synapses, with both serving to potentiate synaptic transmission. As with classical LTP, this form of disinhibition-mediated potentiation was found to be dependent upon Ca^2+^ influx via NMDARs [73]. Indeed, other work has shown that activation of NMDARs can lead to a rapid and enduring decrease in KCC2-mediated Cl^−^ transport [87], while NMDAR signalling during LTP induction leads to a reduction in the total levels of KCC2 [88]. It has yet to be established whether or not this apparent GABAergic plasticity is relatively synapse specific, as has been reported for glutamatergic LTP, or whether GABAergic inputs are affected across larger parts of the dendrite or indeed across the entire cell. Nevertheless, these findings raise the possibility that the expression of NMDAR-mediated LTP might involve a component of GABAergic plasticity.

Amongst neurological disorders, neural trauma and hyperactivity have been shown to lead to long-term changes in the *E*_GABA_ of the affected neurons. Yet such changes to the ionic driving force for GABA_A_Rs may in turn work to contribute to, or exacerbate, the abnormal activity patterns associated with these pathological states. In a landmark paper investigating the propagation of epileptic activity between two interconnected and intact hippocampi, Khalilov et al. [8] showed that seizure activity in one hippocampus could propagate to the naive hippocampus and eventually transform it into an epileptic structure capable of generating seizures. Subsequent investigation of the *E*_Cl^−^_ of neurons in this secondary epileptic focus revealed that the cells had undergone an excitatory shift in the driving force of their GABA_A_R synapses. Stimulating GABA_A_Rs within the secondary focus resulted in bursts of action potentials in the absence of any glutamatergic signalling, leading the authors to conclude that such excitatory actions of GABA may generate seizures in the newly epileptic tissue [8]. Such shifts in the *E*_GABA_ do not need to be overtly excitatory in order to alter neural circuit activity. In rat dentate granule cells, induction of status epilepticus via *in vivo* pilocarpine injections can lead to depolarising *E*_GABA_ and impaired Cl^−^ extrusion capabilities [15]. The depolarising GPSPs increased the probability of action potential generation when paired with excitatory inputs and compromised the ability of the dentate gyrus to filter inputs from the entorhinal cortex [15]. 

The long-term and short-term changes to *E*_GABA_ observed in pathological states, or following pathological activity patterns, can be considered as relatively large changes, often switching the driving force of GABA_A_Rs from hyperpolarising to depolarising and even excitatory [8, 10, 15, 60–62, 70]. By contrast, changes to [Cl^−^]*_i_* following what could be considered more physiologically normal spiking activity typically result in much smaller modifications to the driving force for GABA_A_Rs, usually within the range of approximately 3–10 millivolts. Given these relatively modest shifts an important question is to what extent such plasticity might alter subsequent activity in the affected cells. Artificially setting the *E*_GABA_ of a neuron is one way of exploring how changes to the ionic driving force for GABA_A_Rs may impact activity. This has been achieved experimentally by either altering [Cl^−^]*_i_* via intracellular dialysis of different Cl^−^ concentrations during whole-cell recordings, or by simulating GABAergic inputs with different *E*_GABA_ values using the dynamic-clamp recording configuration. In several studies that have adopted these approaches, shifting *E*_GABA_ to depolarising values led to increased neuronal excitability, resulting in enhanced spiking probability and reduced spike latencies in response to GABAergic inputs, as well as facilitation of voltage-sensitive NMDAR transmission [89–92]. Another approach which has made it possible to explore the functional impact of relatively small changes in [Cl^−^]*_i_* and *E*_GABA_ has been computational modelling. These studies have shown that modest shifts in *E*_GABA_ can have a significant impact on neural signalling. For example, changing the *E*_GABA_ in a model of a mature CA1 pyramidal neuron from −75 mV to −70 mV (a similar level of long-term depolarising shift to that observed experimentally) results in an increase in action potential firing frequency by approximately 40% [92]. Likewise, positive shifts in inhibitory reversal potentials by as little as 10 mV can markedly shorten the duration of inhibitory inputs within the soma [33]. Changes to neural output resulting from modest shifts in *E*_GABA_ can be further exaggerated depending on other factors such as the frequency and location of GABAergic inputs [33, 93]. For instance, in neonatal spinal cord, GPSPs are depolarising but still mediate inhibitory effects via shunting actions. In computational models of these neurons, moving the *E*_Cl^−^_ to more depolarised values reduces the time window over which GPSPs exert functional inhibition of excitatory activity within the soma, particularly when the shift in *E*_Cl^−^_ occurs at distal inhibitory inputs so that shunting effects associated with the GABAergic conductance have less impact [33].

Modest changes to *E*_GABA_ are likely to be especially significant when the balance between GABA_A_R inhibition and facilitation is a fine one. For example, in neocortical layer 5 pyramidal neurons *E*_GABA_ has been calculated to lie at values more depolarising than the resting membrane potential, but below the action potential threshold [94]. Depending on their timing in relation to glutamatergic inputs, somatic GABA_A_R inputs can either shunt or facilitate excitatory inputs, which can impose a bidirectional modulation on neuronal firing rates [94]. By simulating different timing relationships between GABAergic and glutamatergic inputs in a model neocortical neuron, Morita et al. [95] showed that such bidirectional modulation of firing rates by GABA_A_Rs was possible when the *E*_GABA_ lies within a narrow range of values close to the original *E*_GABA_ value calculated by Gulledge and Stuart [94]. Increasing *E*_GABA_ by only a few millivolts was enough to severely reduce the relative timing window in which GABA_A_R inputs could have an inhibitory effect upon neuronal firing rate compared to a facilitating one. Moving *E*_GABA_ more negative by a few millivolts, such that it was equal to the resting membrane potential of the model cell, was sufficient to render GABA_A_R inputs completely inhibitory, regardless of their relative timing to glutamatergic inputs [95]. Similarly, it has been shown that when *E*_GABA_ falls within a specific voltage range, GPSPs can have a bidirectional effect on spike times in visual cortex—either delaying or advancing the time of spikes relative to oscillatory changes in membrane potential [96]. As precise spike timing has been widely implicated in neural processing [97–99] and synaptic plasticity [97, 98], the alterations in spiking activity brought on by small shifts in *E*_GABA_ may therefore have important consequences for information coding and brain development.

## 5. Summary

In summary, the driving force for GABA_A_Rs should not be considered a fixed parameter underlying fast synaptic inhibition, but rather a dynamic parameter, that exhibits both spatial and activity-dependent modulation. The concept that *E*_GABA_ changes in the context of neural development and certain neuropathological conditions is well established. However, more recent studies in this area have revealed that neurons have a range of sophisticated mechanisms for regulating the ionic driving force for GABA_A_Rs. *E*_GABA_ has been reported to vary between different cellular compartments and may even exhibit synapse-specific variation within a single neuron. In addition, the driving force for GABA_A_Rs can be changed “on the fly” and is subject to both short- and long-term temporal changes via a range of activity-dependent mechanisms. These processes are further subject to developmental regulation, where changes in activity patterns can target different regulators of [Cl^−^]*_i_* and drive *E*_GABA_ in different directions depending on the age of the neuron. Further dissecting the mechanisms that regulate such a fundamental aspect of GABAergic transmission should improve our understanding of synaptic integration mechanisms in both health and disease.
